# Across-frequency behavioral estimates of the contribution of inner and outer hair cell dysfunction to individualized audiometric loss

**DOI:** 10.3389/fnins.2014.00214

**Published:** 2014-07-23

**Authors:** Peter T. Johannesen, Patricia Pérez-González, Enrique A. Lopez-Poveda

**Affiliations:** ^1^Auditory Computation and Psychoacoustics, Instituto de Neurociencias de Castilla y León, University of SalamancaSalamanca, Spain; ^2^Grupo de Audiología, Instituto de Investigación Biomédica de Salamanca, University of SalamancaSalamanca, Spain; ^3^Departamento de Cirugía, Facultad de Medicina, Facultad de Medicina, Universidad de SalamancaSalamanca, Spain

**Keywords:** cochlear non-linearity, auditory masking, hearing aid, cochlear damage, hearing loss, hearing impairment

## Abstract

Identifying the multiple contributors to the audiometric loss of a hearing impaired (HI) listener at a particular frequency is becoming gradually more useful as new treatments are developed. Here, we infer the contribution of inner (IHC) and outer hair cell (OHC) dysfunction to the total audiometric loss in a sample of 68 hearing aid candidates with mild-to-severe sensorineural hearing loss, and for test frequencies of 0.5, 1, 2, 4, and 6 kHz. It was assumed that the audiometric loss (HL_TOTAL_) at each test frequency was due to a combination of cochlear gain loss, or OHC dysfunction (HL_OHC_), and inefficient IHC processes (HL_IHC_), all of them in decibels. HL_OHC_ and HL_IHC_ were estimated from cochlear I/O curves inferred psychoacoustically using the temporal masking curve (TMC) method. 325 I/O curves were measured and 59% of them showed a compression threshold (CT). The analysis of these I/O curves suggests that (1) HL_OHC_ and HL_IHC_ account on average for 60–70 and 30–40% of HL_TOTAL_, respectively; (2) these percentages are roughly constant across frequencies; (3) across-listener variability is large; (4) residual cochlear gain is negatively correlated with hearing loss while residual compression is not correlated with hearing loss. Altogether, the present results support the conclusions from earlier studies and extend them to a wider range of test frequencies and hearing-loss ranges. Twenty-four percent of I/O curves were linear and suggested total cochlear gain loss. The number of linear I/O curves increased gradually with increasing frequency. The remaining 17% I/O curves suggested audiometric losses due mostly to IHC dysfunction and were more frequent at low (≤1 kHz) than at high frequencies. It is argued that in a majority of listeners, hearing loss is due to a common mechanism that concomitantly alters IHC and OHC function and that IHC processes may be more labile in the apex than in the base.

## Introduction

Cochlear hearing loss occurs when absolute hearing thresholds for pure tones are higher than normal without signs of middle-ear or auditory neural pathology (Moore, 2007). In the healthy cochlea, inner hair cells (IHCs) transduce mechanical basilar membrane (BM) vibrations into nerve signals, while outer hair cells (OHCs) amplify BM responses to low-level sounds and are thus responsible for our high auditory sensitivity (Bacon et al., 2004). A reduction in the number of OHCs or lesions to the OHCs or associated structures can reduce the cochlear gain to low level sounds and hence cause an audiometric loss. Similarly, a reduction in IHC count or lesions to the IHCs or their associated structures can increase the BM excitation required for detecting a signal, which may also cause an audiometric loss (Moore, 2007). Although it is not generally possible to establish a one-to-one correspondence between audiometric loss and the degree of physical IHC/OHC loss or injury (Chen and Fechter, 2003; Lopez-Poveda and Johannesen, 2012), it is reasonable to assume that the audiometric loss may be due to combined loss or dysfunction of IHCs and OHCs. Indeed, some authors have assumed that the audiometric loss (HL_TOTAL_) for a given test frequency may be conveniently expressed as the sum of two contributions: one associated with cochlear mechanical gain loss, or OHC dysfunction (HL_OHC_), and one associated with inefficient IHC transduction, or IHC dysfunction (HL_IHC_), where HL_TOTAL_, HL_IHC_ and HL_OHC_ are all in decibels (dB) (Moore and Glasberg, 1997; Plack et al., 2004; Moore, 2007; Jepsen and Dau, 2011; Lopez-Poveda and Johannesen, 2012). The aim of the present study was to assess HL_OHC_ and HL_IHC_ over the frequency range from 500 Hz to 6 kHz in a large sample of listeners with mild-to-severe sensorineural hearing loss.

The prevailing view is that OHCs are generally more labile than IHCs and that IHCs and OHCs in the basal region of the cochlea are damaged first and to a greater extent than cells in the apical region (reviewed by Møller, 2000). The relative degree of physical IHC/OHC loss or dysfunction and the location of the dysfunction, however, almost certainly depend on the cause and magnitude of the lesion. Noise-induced hearing loss is associated mostly with loss of basal OHCs (Chen and Fechter, 2003). In human, temporal bone studies of noise-induced hearing loss report increased cell death in basal BM locations and fewer surviving OHCs than IHCs (McGill and Schuknecht, 1976). On the other hand, acoustic trauma damages IHC and OHC stereocilia to similar degrees, which suggests that noise-induced hearing loss probably has a substantial contribution from IHC dysfunction (Liberman and Dodds, 1984). The cochlear location of the dysfunction almost certainly depends on the noise spectrum.

Some ototoxic drugs also cause a hearing loss. In this case, the degree of physical IHC and OHC damage depends on the drug employed. Aminoglycosides cause mostly OHC dysfunction and basal OHCs are first affected and more affected than apical OHCs (van Ruijven et al., 2004; Selimoglu, 2007; Pickles, 2008). Carboplatin, by contrast, does not reduce otoacoustic emission levels (Trautwein et al., 1996) or the sharpness of neural response tuning curves (Wang et al., 1997), which suggests that carboplatin hardly affects cochlear mechanics and affects mostly IHCs or their related structures. Furthermore, carboplatin raises the tips of neural tuning curves comparably at all frequencies (Wang et al., 1997), which indicates that its effect on IHCs is comparable along the cochlear length. In humans, histological studies of aminoglycoside-induced hearing loss report increased cell death in basal BM location and fewer surviving OHCs than IHCs (Huizing and de Groot, 1987).

Sensorineural hearing loss, however, need not always be caused by reduced counts or injury to hair cells or their associated structures. Metabolic presbycusis, for example, a form of age-related hearing loss (Schmiedt et al., 2002), causes a reduction of the endocochlear potential that can simultaneously reduce the cochlear mechanical gain (Saremi and Stenfelt, 2013) *and* the IHC response (Meddis et al., 2010; Panda et al., 2014) Functionally, this can manifest as a simultaneous dysfunction of IHCs and OHCs. Computer simulation studies suggest that whatever the mechanism, a reduction of the endocochlear potential always raises absolute thresholds more at high than at low frequencies (Meddis et al., 2010; Saremi and Stenfelt, 2013; Panda et al., 2014), which probably explains the association between aging and gradually sloping high-frequency losses. Likewise, aspirin, an ototoxic agent that impairs OHC function, broadens psychoacoustical tuning curves, reduces two-tone suppression, and linearizes growth-of-masking functions slightly more at 3 kHz than at 750 Hz, which can be explained in terms of greater involvement of labile cochlear non-linear processes in basal than in apical cochlear regions (Hicks and Bacon, 1999). In summary, it would be erroneous to conclude that the typically greater high-frequency losses are always due to comparatively greater loss or injury of basal than apical IHCs and/or OHCs.

Regardless of its actual cause, sensorineural hearing loss is typically treated with hearing aids. In programming a hearing aid, the assumption is made that HL_TOTAL_ is partly due to cochlear mechanical gain loss (akin to HL_OHC_) and partly due to other factors (akin to HL_IHC_). Individual across-frequency estimates of HL_OHC_ and HL_IHC_ would be highly useful to optimize individualized treatment with hearing aids (Muller and Janssen, 2004; Mills, 2006). Estimation of HL_OHC_ and HL_IHC_ is, however, hard because it can only be done using indirect methods. For this reason, large-scale studies are rare. Using a loudness model, Moore and Glasberg (1997) concluded that HL_OHC_ and HL_IHC_ account on average for 80 and 20% of HL_TOTAL_, respectively, but reported that for a few listeners the loss attributable to OHC damage appears to be less than 50%. Plack et al. (2004) used the temporal masking curve (TMC) method (Nelson et al., 2001) to infer I/O curves at 4 kHz and estimated that HL_OHC_ contributes 65% of HL_TOTAL_. Also based on TMC data, Jepsen and Dau (2011) used a computer auditory model to estimate HL_IHC_ and HL_OHC_ at 1 and 4 kHz in 10 hearing impaired (HI) listeners. Their results were broadly consistent with the common view that HL_OHC_ is greater and more frequent than HL_IHC_, but they also reported some cases with substantial HL_IHC_ at low frequencies. Jürgens et al. (2011) concluded that at 4 kHz cochlear gain loss (or HL_OHC_) was proportional to HL_TOTAL_ but 10–15 dB lower (p. 189). More recently, we have proposed a more refined method for estimating HL_OHC_ and HL_IHC_ from the analysis of TMC-based input/output (I/O) curves. We concluded that HL_OHC_ and HL_IHC_ account on average for 60 and 40% of HL_TOTAL_ with large variability across cases; indeed, percentages were sometimes reversed (Lopez-Poveda and Johannesen, 2012). Our conclusions were based on 26 I/O curves (most of them for a test frequency of 4 kHz) from 18 listeners with mild-to-moderate sensorineural hearing losses and are awaiting confirmation and extension to other frequencies and broader range of hearing losses.

The main aim of the present study was to assess HL_IHC_ and HL_OHC_ from behaviorally inferred I/O curves for a large sample of hearing aid candidates (*N* = 68) and for test frequencies of 0.5, 1, 2, 4, and 6 kHz. A second objective was to investigate to what extent HL_IHC_ and HL_OHC_ vary across test frequencies to examine potential structure-function correlations; that is, to examine the potential correspondence between HL_OHC_ and HL_IHC_ with existing evidence regarding physical loss or injury and/or dysfunction of OHCs and IHCs and their distribution across frequency. A third objective was to investigate the degree of variability of HL_OHC_ and HL_IHC_ across listeners. We used virtually the same approach as in our recent study (Lopez-Poveda and Johannesen, 2012). The present work, however, extends our previous study in several important aspects: first, HL_OHC_ and HL_IHC_ estimates in our previous study were restricted to I/O curves that showed a “knee-point” or a compression threshold (CT), whereas the present analysis is extended to all I/O curves; second, the present study is for a much larger subject sample and for a wider range of frequencies; third, the present study included participants with hearing losses from mild to severe, hence more representative of the hearing-aid candidate population.

## Methods

### Approach and assumptions

Our approach was virtually identical to that of Lopez-Poveda and Johannesen (2012). The details can be found in that publication and for conciseness only a summary is provided here. After Moore and Glasberg (1997), we assumed that the total audiometric loss may be split into two contributions: one pertaining to a reduction of mechanical cochlear gain due to OHC dysfunction and a remaining component, which, for convenience, will be assumed due to inefficient IHC processes, or IHC dysfunction:

(1) $${\text{HL}}_{\text{TOTAL}}={\text{HL}}_{\text{OHC}}+{\text{HL}}_{\text{IHC}},$$

where HL_TOTAL_, HL_OHC_, and HL_IHC_ are all in dB. In what follows, HL_OHC_ and HL_IHC_ will be referred to as “OHC loss” and “IHC loss,” respectively, and should be interpreted as contribution to audiometric loss (in dB) rather than as anatomical lesions or reduced cell counts.

We further assumed that HL_OHC_ can be found using the OHC dysfunction model of Plack et al. (2004). In this model, a cochlear mechanical input/output (I/O) curve is modeled by a function consisting of a linear segment (slope ~1 dB/dB) at low input levels, followed by a compressive segment at mid-level inputs (slope < 1 dB/dB), eventually followed by another linear segment at high input levels. The breakpoint between the low-level linear segment and the compressive segment is referred to as the CT and the breakpoint between the mid-level compressive segment and the high-level linear segment is referred to as the return-to-linearity threshold (RLT). OHC dysfunction causes a loss of low-level cochlear gain and is modeled as a horizontal shift of the low-level linear segment of the I/O curve toward higher input levels without affecting the slope of the compressive segment (Plack et al., 2004). An assumption of our approach is that HL_OHC_ can be found by comparing the CT of a given hearing-impaired (HI) listener with a reference CT for normal hearing (NH) listeners (Lopez-Poveda and Johannesen, 2012). When an HL_OHC_ estimate is available, then HL_IHC_ can be estimated using Equation (1) as the difference between HL_OHC_ and HL_TOTAL_. For a sufficiently large OHC dysfunction, all the cochlear gain is lost, the I/O curve becomes linear (absent CT) and HL_OHC_ is assumed to be equal to the NH gain.

IHC dysfunction is assumed to increase the BM excitation needed for signal detection at threshold. When estimating the I/O curve with a psychophysical approach, only the part of the I/O curve that is above the cochlear mechanical excitation required for detection can be measured. For a large increase in BM excitation, a CT may be absent and only a part of the compressive portion of the I/O curve is available. Therefore, the absence of a CT with presence of a compressive segment in the I/O curve is assumed as indicative of substantial HL_IHC_. For these cases, it is assumed that Equation (1) does not hold and that HL_TOTAL_ ~ HL_IHC_. In other words, it is assumed that even though HL_OHC_ may occur, it does not contribute to the audiometric hearing loss (for a full explanation, see Figure 1D in Lopez-Poveda and Johannesen, 2012 and its related text).

Estimation of HL_IHC_ and HL_OHC_ as outlined above requires access to cochlear I/O curves. We assumed that I/O curves can be inferred behaviorally using the TMC method of Nelson et al. (2001). Briefly, this method consists of measuring the levels of a pure tone forward masker required to just mask a following fixed low-level probe tone as a function of the masker-probe time gap. Two TMCs are measured to infer an I/O curve: one for a condition where the masker is processed linearly by the BM (linear reference); and one for a condition where the masker and the probe tones are equal in frequency (on-frequency). It is assumed that the slope of the linear-reference TMC reflects the post-mechanical rate of recovery from forward masking while the slope of the on-frequency TMC reflects both BM compression on the masker *and* the post-mechanical rate of recovery from forward masking. Under the assumption that the post-mechanical rate of recovery is independent of masker level and frequency, a cochlear I/O curve can be inferred by plotting the masker levels of the linear reference TMC as a function of the masker levels for the on-frequency TMC for paired time gaps (Nelson et al., 2001). Lopez-Poveda et al. (2003) proposed to use a common linear-reference TMC for a high-frequency probe and a low-frequency masker to infer I/O curves for all probe frequencies on the assumption that the recovery from forward masking is also independent of the probe frequency.

### Participants

**Figure 1:**
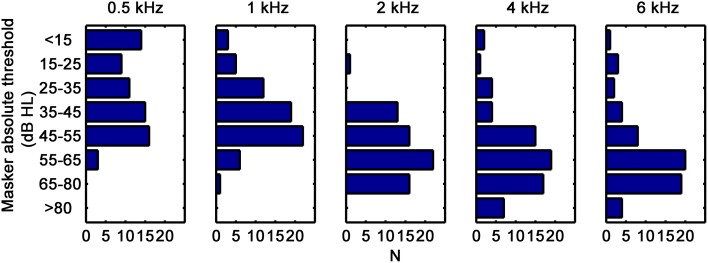
**Distributions of corrected masker absolute thresholds, in dB HL (see text for details)**. Each panel is for a different test frequency, as indicated at the top.

A total of 68 listeners (43 males) with symmetrical sensorineural hearing loss participated in the study. Their ages ranged from 25 to 82 years (median = 61 years). Air conduction absolute thresholds were measured using a clinical audiometer (Interacoustics AD229e) at the typical audiometric frequencies (0.125, 0.25, 0.5, 1, 2, 3, 4, 6, and 8 kHz) (ANSI, 1996). Bone conduction thresholds were measured at 0.5, 1, 2, 3, and 4 kHz. Air and bone conduction thresholds were also measured at 0.75 and 1.5 kHz for a large subset of subjects. A hearing loss was regarded as sensorineural when tympanometry was normal and air-bone gaps were smaller than or equal to 15 dB at one frequency and smaller than or equal to 10 dB at any other frequency. Participants were recruited for a large-scale bilateral hearing-aid outcome study. Therefore, they were additionally required to be hearing-aid candidates (as judged by an experienced audiologist) and to have symmetrical bilateral loss. A hearing loss was regarded as symmetrical when the mean air conduction thresholds at 0.5, 1, and 2 kHz differed by less than 15 dB between the two ears, and the mean difference at 3, 4, and 6 kHz was less than 30 dB (AAO-HNS, 1993). For the current purpose, each participant was tested in one ear. The ear was selected to maximize the number of test frequencies for which TMCs could be obtained. For the majority of cases, this meant selecting the ear with better thresholds in the 2–6 kHz frequency range (30 left ears, 38 right ears). Figure 1 gives an idea of the distribution of hearing losses (see below).

The data from our previous related study was also included in the present analysis (Lopez-Poveda and Johannesen, 2012). This included reference data for 15 NH listeners and data for 18 listeners with mild-to-moderate sensorineural hearing loss. Results for these groups will be clearly identified below.

All procedures were approved by the human experimentation ethical review board of the University of Salamanca. Subjects gave their signed informed consent prior to their inclusion in the study.

### TMC stimuli and procedure

Stimuli and procedure were similar to those of Lopez-Poveda and Johannesen (2012). On-frequency TMCs were measured for probe frequencies (*f_P_*) of 0.5, 1, 2, 4, and 6 kHz. Maskers and probes were sinusoids. The duration of the maskers was 210 ms including 5-ms cosine-squared onset and offset ramps. Probes had durations of 10 ms, including 5-ms cosine-squared onset and offset ramps with no steady state portion, except for the 500-Hz probe, whose duration was 30 ms with 15-ms ramps and no steady state portion. The level of the probes was fixed at 10 dB above the individual absolute threshold for the probe. Masker-probe time gaps, defined as the period from masker offset to probe onset, ranged from 5 to 100 ms in 10-ms steps with an additional gap of 2 ms. Masker levels sometimes reached the maximum permitted sound level output (105 dB SPL) after a few time gaps. If the number of measured data points was insufficient for curve fitting (see below), masker levels were measured for additional intermediate gaps (e.g., 5, 15, 25 ms). In a few cases, masker levels were atypically low for a time gap of 100 ms. In these cases, masker levels were measured for additional gaps in the range 110–140 ms.

A single linear reference TMC was measured for each listener and it was used to infer I/O curves for all other probe frequencies (Lopez-Poveda et al., 2003). The linear reference TMC was for a probe frequency of 2, 4, or 6 kHz and for a masker frequency equal to 0.4*f_p_* or 0.5*f_p_*. The selection of linear reference condition depended on the listener's hearing loss at the linear-reference probe frequency and on the maximum permitted sound level output (105 dB SPL). Following the indications of earlier studies, (Lopez-Poveda et al., 2003; Lopez-Poveda and Alves-Pinto, 2008), the linear reference conditions were sought in the order of priority shown in Table 1.

**Table 1:** **Prioritized linear-reference TMC conditions and number of cases (*N*) where each condition applied (see also red curves in Figure 2)**.

**Priority order**	**①**	**②**	**③**	**④**	**⑤**	**⑥**
Probe (kHz)	4	4	6	6	2	2
Masker (kHz)	1.6	2	2.4	3	0.8	1
*N*	34	6	4	4	9	6

*Note that these numbers add up to 63 rather than to the total number of participants (N = 68). This is because a linear reference TMC could not be measured for four participants, and the data from one additional participant who performed inconsistently during the task were excluded from the analysis*.

Stimuli were generated digitally in Matlab and output via an RME Fireface 400 sound card (sampling frequency of 44100 Hz, 24-bit resolution) and delivered to the listeners through Sennheiser HD-580 headphones. Sound pressure levels (SPL) were calibrated by placing the headphones on a KEMAR equipped with a Zwislocki DB-100 artificial ear connected to a sound level meter. Calibration was performed at 1 kHz only and the obtained sensitivity was used at all other frequencies.

Masker levels at threshold were measured using a two-interval, two-alternative, forced-choice adaptive procedure with feedback. The inter-stimulus interval was 500 ms. The initial masker level was set sufficiently low that the listener always could hear both the masker and the probe. Masker level was then changed according to a two-up, one-down adaptive procedure to estimate the 71% point on the psychometric function (Levitt, 1971). An initial step size of 6 dB was applied, which was decreased to 2 dB after three reversals. The adaptive procedure continued until a total of 12 reversals in masker level were measured. Threshold was calculated as the mean masker level at the last 10 reversals. A measurement was discarded if the standard deviation of the last 10 reversals exceeded 6 dB. Three threshold estimates were obtained in this way and their mean was taken as the threshold. If the standard deviation of these three measurements exceeded 6 dB, one or more additional threshold estimates were obtained and included in the mean. Measurements were made in a double-wall sound attenuating booth. Listeners were given at least 2 h of training on the TMC task before data collection began.

Absolute thresholds for the probes and maskers were measured using a similar procedure except that the adaptive procedure was one-up, two-down.

TMC and absolute threshold measurements took between 12 and 15 h per participant in total and were distributed in several (1- or 2-h) sessions on several days.

### TMC fitting

Linear-reference and on-frequency TMCs were fitted before they were used to infer I/O curves. Linear reference TMCs were fitted with a double exponential function with four parameters (Lopez-Poveda and Johannesen, 2012); on-frequency TMCs were fitted with a function consisting of the double exponential function fitted to the linear reference TMC plus a second-order Boltzmann function with six parameters (Lopez-Poveda and Johannesen, 2012). When fitting the on-frequency TMC, the parameters of the double exponential function were held fixed and only the parameters of the second-order Boltzmann function were allowed to vary. When the number of data points in a TMC was equal or fewer than the number of parameters of the double exponential or the second-order Boltzmann function, single exponential (two parameters) and first-order (four parameters) Boltzmann functions were used instead. A full justification of this approach can be found elsewhere (Lopez-Poveda and Johannesen, 2012). The goodness-of-fit was assessed using the root-mean-square (RMS) error between measured and fitted TMCs. RMS errors were less than 2 dB for all linear reference TMCs, and less than 4 dB for on-frequency TMCs, except for three cases for which RMS errors were less than 6 dB.

### Inference of I/O curves

I/O curves were inferred for each participant by plotting the masker levels of his/her linear reference TMC against the masker levels for the on-frequency TMCs paired according to time gaps (Nelson et al., 2001). For any given participant, a common linear reference condition was used to infer I/O curves at all test frequencies (Lopez-Poveda et al., 2003). A linear reference TMC could not be found for four participants because their hearing loss was too high at the linear-reference probe frequencies (Table 1). In these four cases, an average linear reference (mean across all other participants for the condition *f_P_* = 4 kHz and *f_m_* = 1.6 kHz) was used to infer I/O curves. This average linear reference TMC was also used for reanalysis of four cases from our previous study (Lopez-Poveda and Johannesen, 2012) that did not have linear reference for the same reason.

## Results

### Hearing loss distributions

Absolute thresholds for the maskers were used to assess hearing losses. Masker duration was shorter for the present participants (200 ms) than for the NH reference group or the HI listeners used in our previous study (300 ms in Lopez-Poveda and Johannesen, 2012). Because absolute threshold depends on signal duration, this difference in masker duration could have introduced a small difference in threshold for the participants in each study. Given that HL_TOTAL_ was defined as the difference between masker thresholds of the HI and NH listeners, an attempt was made to correct the present masker thresholds for the influence of duration on absolute thresholds by adding the difference between NH absolute thresholds for pure tone durations of 300 and 200 ms to the masker thresholds of the present HI subjects (Watson and Gengel, 1969). Corrections were smaller than 1 dB at all frequencies. Figure 1 shows the corrected absolute thresholds for the present participants; thresholds for the HI participants from our previous study are omitted in Figure 1 but can be found in the original reference. Clearly, on average, participants had high-frequency losses typical of presbycusis but the range of hearing losses at each frequency was quite variable.

### Temporal masking curves

**Figure 2:**
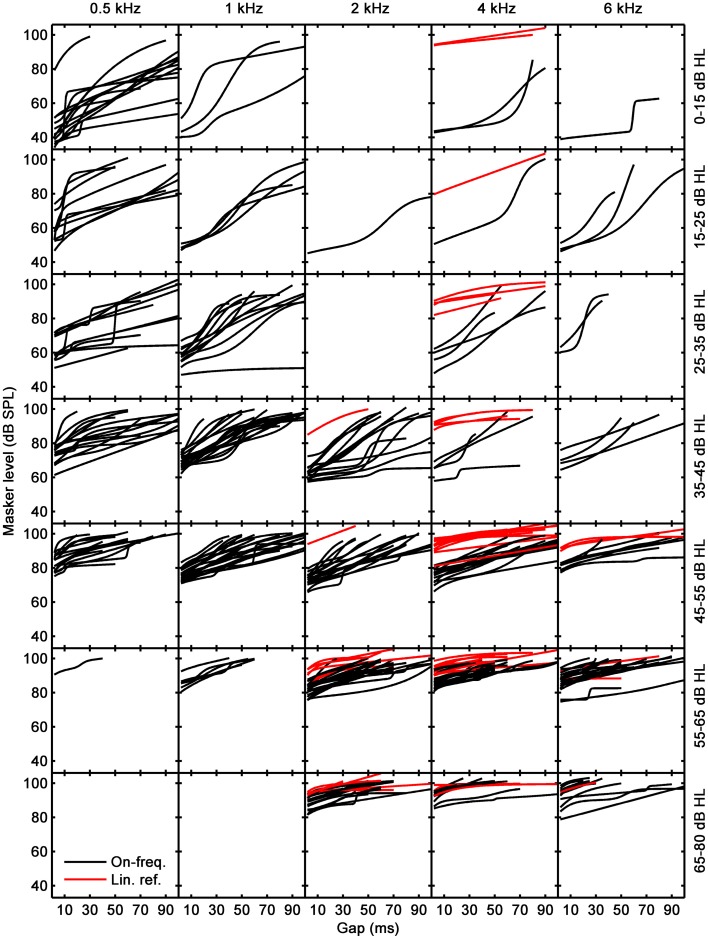
**Fitted linear-reference (red curves) and on-frequency TMCs (black curves)**. Each column corresponds to a different test frequency as indicated by the column title and each row to different a hearing-loss range, as indicated by the text on the right-most ordinate.

Figure 2 shows fitted linear-reference and on-frequency TMCs for 67 participants; one participant performed inconsistently during the TMC task and her data were excluded from further analysis. Measured TMCs are omitted in Figure 2 to avoid clutter. Each column is for a different test frequency as indicated by column title, and each row is for a hearing-loss range as indicated by the text on the right-most ordinate. Both linear-reference (red curves) and on-frequency TMCs (black curves) had characteristics similar in most aspects to those published in earlier reports (Nelson et al., 2001; Plack et al., 2004; Lopez-Poveda et al., 2005; Jepsen and Dau, 2011; Lopez-Poveda and Johannesen, 2012). The on-frequency masker levels for the shortest time gap (2 ms) decreased with decreasing frequency and on-frequency and linear-reference TMCs were less parallel (i.e., on-frequency TMCs were steeper than linear-reference TMCs) at lower than at higher frequencies. Both these aspects are consistent with listeners having less hearing loss (Figure 1) and presumably less gain loss and more compression at low than at high frequencies.

Lopez-Poveda and Alves-Pinto (2008) argued that an ideal linear-reference TMC for inferring I/O curves would be for *f_p_* = 4 kHz and *f_m_* = 1.6 kHz on the grounds that the slope of such a TMC would be unlikely affected by cochlear compression and would reflect only the post-mechanical rate of recovery from forward masking. As explained above, the hearing loss of some listeners was so large at 4 kHz that it was not possible to measure this preferred linear-reference TMC and alternative linear references were measured instead (Table 1). Before using these linear references to infer I/O curves, we verified that their slopes were statistically comparable to the slopes of the preferred linear reference. To do it, we calculated the mean slope of all measured linear reference TMCs across all available time gaps (red curves in Figure 2), and compared the mean slope of the preferred linear reference condition (denoted as priority ① in Table 1) with the mean slope for every other condition (denoted as priority orders ② to ⑥ in Table 1) using a Student's *t*-test. The tests confirmed that all linear references had statistically equivalent slopes (*p* > 0.05). The difference for conditions ① and ⑤ was close to being significant (*p* = 0.055) but did not reach significance. Therefore, we concluded that all linear-reference TMCs had statistically comparable slopes and that it was reasonable to use them to infer I/O curves.

### I/O curves inferred from TMCs

**Figure 3:**
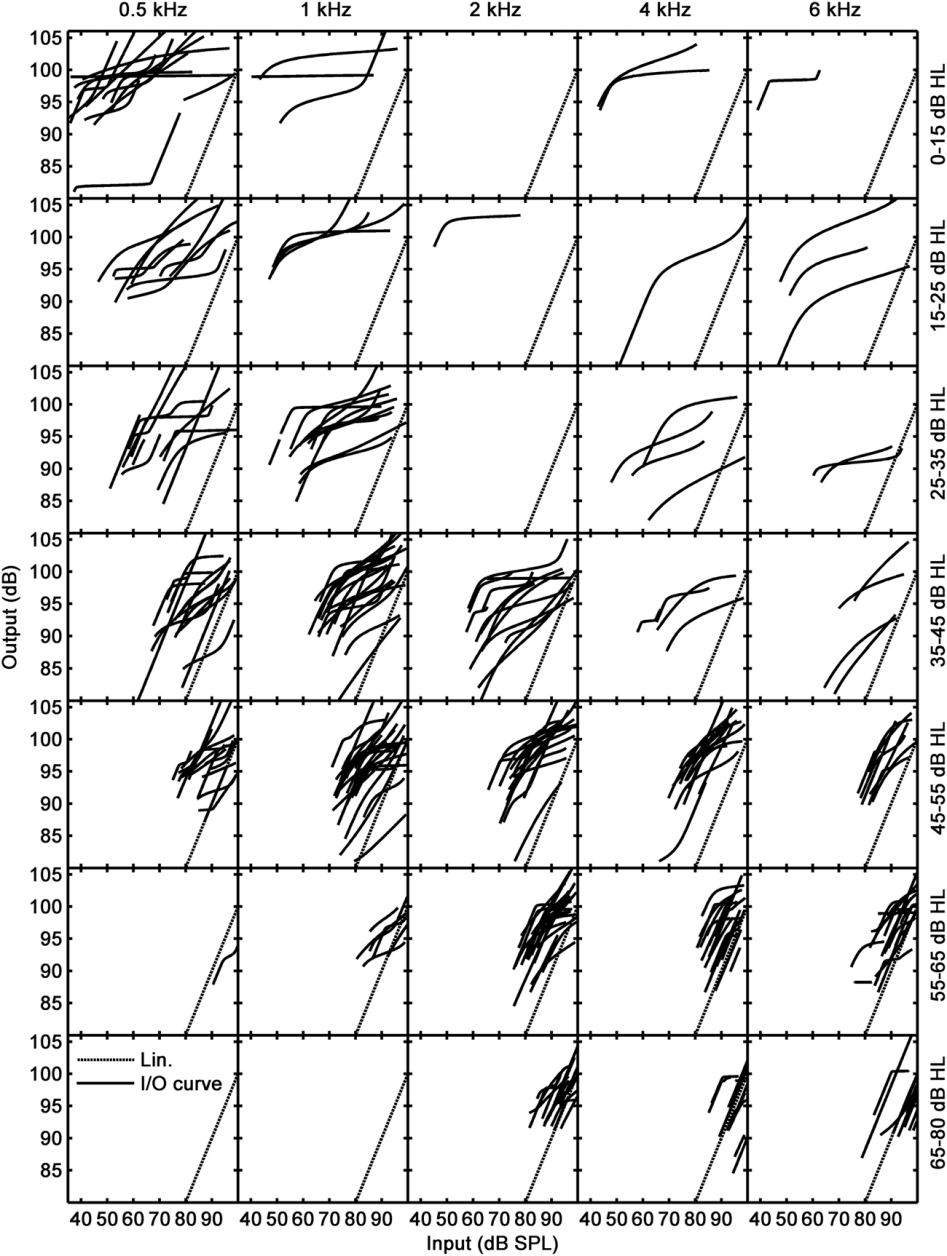
**Inferred I/O curves**. Columns and rows are as in Figure 2.

Figure 3 shows the I/O curves inferred from the TMCs of Figure 2. Dotted lines depict linearity with no gain (input level = output level). The large majority of I/O curves had shapes typical of HI subjects: they often had a linear segment at low input levels followed by a compressive segment at mid input levels, followed sometimes by another linear segment at high input levels. Other I/O curves were best described by an almost straight line with either a compressive slope or with a slope close to linearity. Few I/O curves showed unusual characteristics. For example, their RLTs were surprisingly low (50–70 dB SPL), particularly at low frequencies. Also, some I/O curves were almost flat (e.g., at 1 kHz for hearing loss below 15 dB HL). The latter occurs because their corresponding linear reference TMCs were very shallow. Overall, I/O curves extended to lower input levels at low than at high frequencies, a reasonable result considering that on average participants had greater hearing losses for high than for low frequencies (Figure 1).

### I/O curve analyses and taxonomy

**Figure 4:**
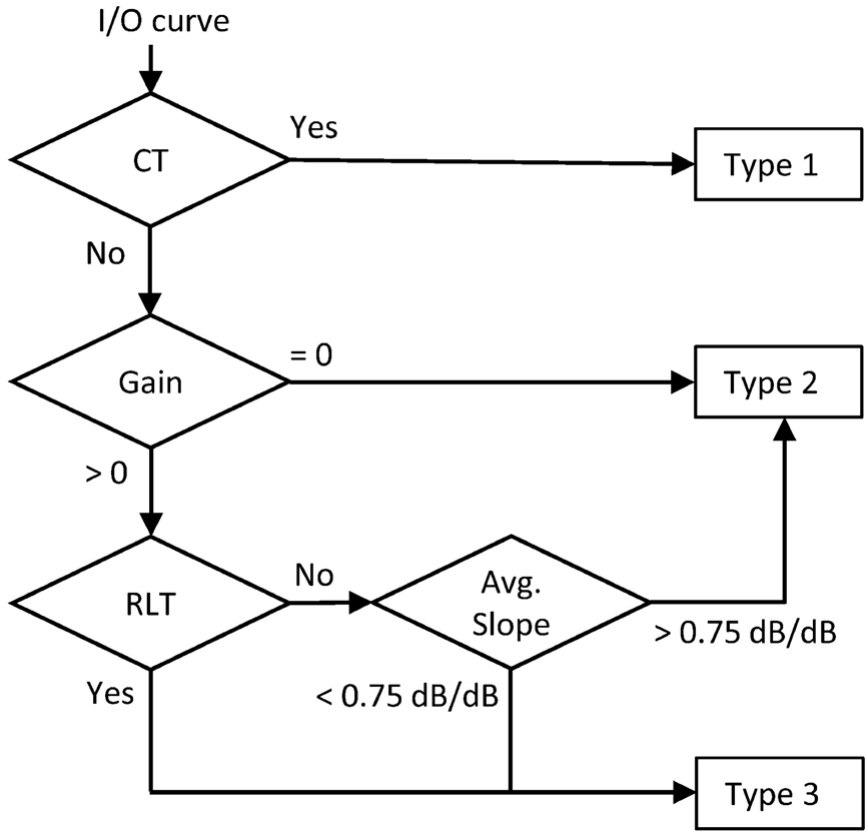
**Flow-diagram and logic used to classify each I/O curve into one of three categories: Type 1 = I/O curve with a compression threshold; Type 2 = Linear I/O curve; Type 3 = I/O curve with residual compression but no compression threshold**.

Lopez-Poveda and Johannesen (2012) argued that HL_OHC_ and HL_IHC_ may be reliably obtained from an I/O curve only if the I/O curve in question shows a CT. They nonetheless hinted that the shape of the I/O curves may be indicative of the type and extent of HL_OHC_ or HL_IHC_ (see their Figure 1 and related text). Here, each I/O curve was analyzed in search for HL_OHC_ and HL_IHC_ using their reasoning and following the logic outlined in Figure 4.

**Figure 5:**
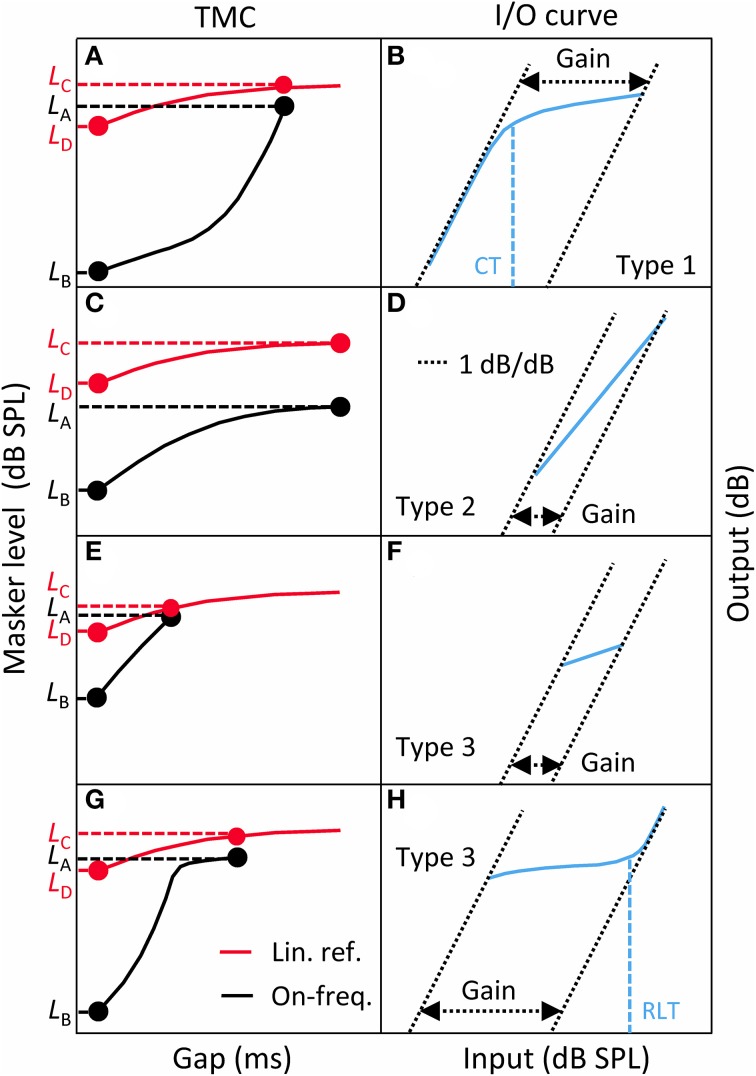
**A taxonomy of I/O curves (blue line, right panels) and their corresponding TMCs (left panels)**. Left: linear-reference (red curves) and on-frequency (black curves) TMCs. Right: corresponding, inferred I/O curves. **(A,B)** for Type 1 I/O curves. **(C,D)** for Type 2 I/O curves. **(E,F)** for Type 3 I/O curves with little residual gain. **(G,H)** for Type 3 I/O curves with large residual gain. CT, compression threshold; RLT, return-to-linearity threshold. See main text for details.

A CT was first sought for each I/O curve. Lopez-Poveda and Johannesen (2012) arbitrarily defined the CT as the input level where the I/O curve reached a slope of 0.5 dB/dB from a higher value at lower input levels (Figure 5B). To take into account the experimental TMC variability on the CT estimate, rather than inferring the CT from the mean I/O curve, they simulated 100 I/O curves for each condition using a Monte-Carlo approach and used the median CT of those simulations in their subsequent analysis. They regarded the obtained CT as unreliable when it was the lowest input level in the mean I/O curve or if the mean I/O curve slope did not reach the criterion value of 0.5 dB/dB, something infrequent in their data (see Lopez-Poveda and Johannesen, 2012). Here, we first tried to apply their same criteria but found many instances where the resulting CTs were unreliable. To maximize the number of I/O curves with valid CTs, we opted to apply slightly different criteria: (1) that 60% of the Monte-Carlo simulated I/O curves showed a valid CT; *and* (2) that the residual cochlear gain of the mean I/O curve (estimated as described below) was greater than zero. The median CT of the Monte-Carlo simulated I/O curves was taken as the final CT.

A large proportion of I/O curves showed a CT (Table 2). Many other I/O curves, however, were best described as straight lines with varying slopes (as depicted in Figure 5D or Figure 5F) or showed a compressive segment and an RLT but no CT (as shown in Figure 5H). The distinction between these cases was made based on residual gain and mean slope using the logic depicted in Figure 4.

**Table 2:** **Number of I/O curves according to their shapes**.

**Frequency (kHz)**	**0.5**	**1**	**2**	**4**	**6**
Type 1 (CT present)	29	54	46	38	24
Type 2 (Linear)	13	4	17	23	22
Type 3 (CT absent with compression)	25	15	7	3	5
Too-high loss	3	2	2	12	20
Total	70	75	72	76	71

*The table includes the present data plus data from (Lopez-Poveda and Johannesen, 2012)*.

Gain was defined here as the difference in sensitivity for low and high input levels, as illustrated in the right panels of Figure 5. That is, gain was defined as the horizontal distance between intersects with the abscissa of two lines with slopes 1 dB/dB that passed through the end points of the I/O curve. Of course, if the measured I/O curve were only a segment of the actual underlying I/O curve, as would happen for instance for straight-line I/O curves like those shown in Figure 5D or Figure 5F, this gain estimate would be smaller than the actual residual gain. Actually, insofar as an I/O curve is inferred from an on-frequency and a linear-reference TMC (compare the left and right panels of Figure 5), gain for all types of I/O curves was directly obtained from the corresponding TMCs as follows:

(2) $$gain=(L_{\text{A}}-L_{\text{B}})-(L_{\text{C}}-L_{\text{D}})\;$$

where *L_A_*, *L_B_*, *L_C_*, and *L_D_* were defined as in Figure 5.

If gain was not significantly different from zero^1^, then the I/O curve was regarded as linear, hence indicative of total gain loss. If, however, gain was greater than zero, we tried to find an RLT in the I/O curve (as shown in Figure 5H). If absent, the I/O curve was regarded as linear when its average slope was steeper than an arbitrary value of 0.75 dB/dB. This criterion prevented cases with small amounts of residual gain and a moderate degree of compression from being erroneously classified as total gain loss; that is, it served to distinguish cases like that shown in Figure 5F, almost certainly indicative of significant IHC dysfunction, from cases like that shown in Figure 5D, almost certainly indicative of total gain loss. If, however, a RLT was present or if the average slope of the I/O curve was <0.75 dB/dB, then we assumed that compression was present and that the I/O curve was indicative of significant IHC dysfunction.

Table 2 shows the number of I/O curves in each of the three categories (Type 1: CT present; Type 2: linear; Type 3: CT absent with compression). The proportion of linear I/O curves was greater at and above 2 kHz than at lower frequencies. The proportion of Type 3 I/O curves was greater at lower than at higher frequencies. In a few cases, the hearing loss was so high (above ~70 dB HL) that measuring the TMC needed to infer an I/O curve would have required masker levels beyond the maximum sound pressure output of our system. These cases, classified as “too-high loss” in Table 2, increased slightly in number with increasing frequency.

Once classified, different I/O curves types were analyzed in search of HL_OHC_ and HL_IHC_. Type 1 I/O curves were analyzed as suggested by Lopez-Poveda and Johannesen (2012); Type 2 and Type 3 I/O curves were analyzed differently, as described below.

### HL_OHC_ and HL_IHC_ estimates from I/O curves

#### From I/O curves with a compression threshold

For I/O curves with a CT (Type 1), HL_OHC_ was calculated as the difference between the CT and the mean CT for the reference NH group multiplied by (1–*c*) (Equation 2 in Lopez-Poveda and Johannesen, 2012), where *c* is the mean compression exponent over the compressive segment of the NH I/O curves. HL_IHC_ was obtained as HL_TOTAL_–HL_OHC_ (Equation 1). This procedure required having mean reference CT and *c* values for NH listeners at each of the test frequencies (0.5, 1, 2, 4, and 6 kHz). Lopez-Poveda and Johannesen (2012) provided reference data for 0.5, 1, and 4 kHz but, to the best of our knowledge, reference data are still lacking at 2 and 6 kHz. For this reason, in the current analysis, the reference values at 4 kHz were used to infer HL_OHC_ and HL_IHC_ also at 2 and 6 kHz. The impact of this approximation on the results is discussed below.

**Figure 6:**
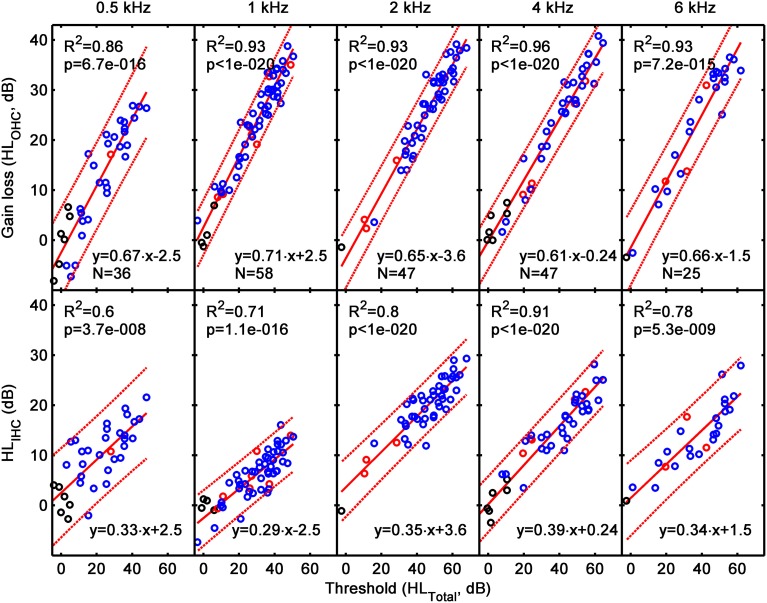
**The contribution of HL_OHC_ (top) and HL_IHC_ (bottom) to HL_TOTAL_ assessed from the analysis of Type 1 I/O curves (i.e., from I/O curves with CT present)**. Each column is a for a different test frequency, as indicated by the column title. Results for the current hearing-impaired listeners are depicted as blue symbols; results for NH listeners and for listeners with mild-to-moderate loss from our earlier study (Lopez-Poveda and Johannesen, 2012) are depicted by black and red symbols, respectively. Continuous lines illustrate mean linear regression functions; dotted lines illustrate 5 and 95% confidence intervals of new individual observations. The insets show linear regression functions and related statistics.

Figure 6 illustrates HL_OHC_ (top) and HL_IHC_ (bottom) as a function of HL_TOTAL_. Note that HL_TOTAL_ is defined here as the difference between a participant's absolute threshold for the masker and the mean absolute masker threshold of the reference NH group (the latter was not 0 dB HL as noted by Lopez-Poveda and Johannesen, 2012). Each column illustrates results for a different test frequency, as indicated at the top of each column. The lower insets in each panel show corresponding linear-regression functions and the number of data points (N) used in the regression; the upper insets show regression statistics, where *R*^2^ is the proportion of variance explained by the regression line, and the *p*-value is the probability of the relationship between the two variables occurring by chance. Red dashed lines depict 95% confidence intervals for a new observation rather than the confidence intervals of the regression lines.

The linear regression functions in Figure 6 show that HL_OHC_ contributed between 61 and 70% to HL_TOTAL_, and HL_IHC_ contributed the rest (30–39%). Interestingly, these percentages were approximately constant across test frequencies, as shown by the slopes of the regression lines. The individual variability of the contributions HL_OHC_ and HL_IHC_ can be assessed from the confidence limits for new single observations. The confidence intervals for HL_OHC_ and HL_IHC_ were around ±9 dB at 0.5 kHz and around ±6 dB over the range 1–6 kHz. In all cases, the confidence intervals were almost independent of the HL_TOTAL_. Recall that these results were only for Type 1 I/O curves.

**Figure 7:**
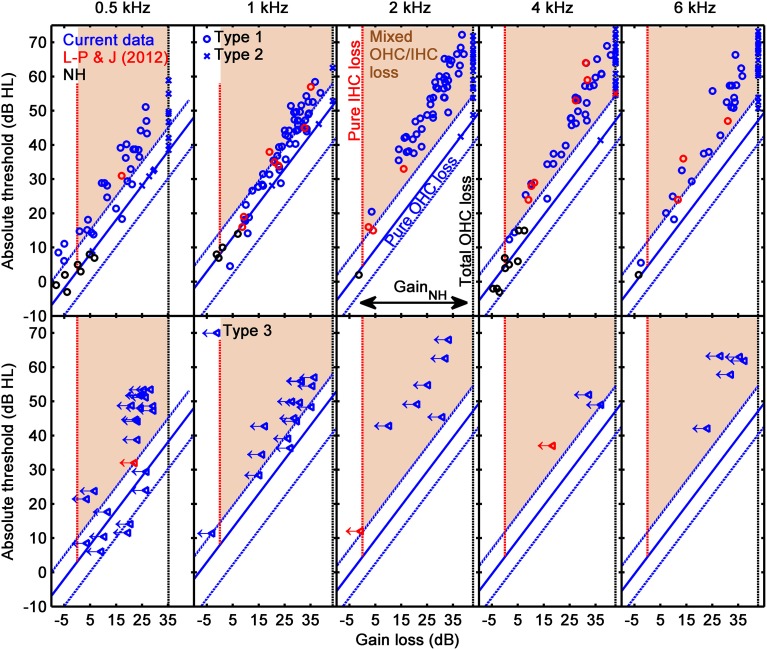
**Absolute threshold as a function of gain loss**. **Top:** results for Type 1 (circles) and Type 2 (crosses) I/O curves. **Bottom panels:** results for Type 3 I/O curves (left-pointing triangles with arrows). Each column is for a different test frequency, as indicated by the column title. In each panel, the diagonal blue line and associated dotted lines indicate mean values and 5% confidence limits for pure OHC loss (HL_TOTAL_ = HL_OHC_), and the vertical black, dotted line depicts the hypothetical location of cases with total cochlear gain loss, as inferred from the I/O curves of the reference, NH sample (black circles) (Johannesen and Lopez-Poveda, 2008). The red dotted lines depict the hypothetical location of cases with pure IHC loss (i.e., hearing loss with zero HL_OHC_). The shaded areas indicate mixed OHC/IHC losses. Results for the current listeners are depicted as blue symbols; results for NH listeners and for listeners with mild-to-moderate loss from our earlier study (Lopez-Poveda and Johannesen, 2012) are depicted as black and red symbols, respectively.

Figure 7 allows statistical judgment of the incidence of cases suffering from pure IHC loss, pure OHC loss, or mixed IHC/OHC loss. The figure illustrates absolute threshold (in dB HL) as a function of cochlear gain loss (HL_OHC_) separately for each frequency. The vertical dotted red line (at HL_OHC_ = 0 dB) indicates the hypothetical location of cases whose hearing loss was exclusively due to IHC dysfunction (pure IHC loss). The blue diagonal line depicts the hypothetical location of cases whose hearing loss was exclusively due to cochlear gain loss (pure OHC loss). The blue-dotted diagonal lines show 5–95% confidence intervals for gain loss as calculated from the reference NH listeners (Lopez-Poveda and Johannesen, 2012). Note that the diagonal does not match with the condition HL_TOTAL_ = HL_OHC_, as one might expect, because as explained by Lopez-Poveda and Johannesen (2012), their NH listeners did not have a mean hearing loss of 0 dB HL. The shaded area indicates the placement of cases whose hearing loss is due partly to cochlear gain loss (HL_OHC_) plus an additional component (mixed OHC/IHC loss). The results from I/O curves with a CT are depicted as blue circles in the top panels of the figure. For completeness, also shown are the results for listeners with NH (black circles) and mild-to-moderate hearing loss (red circles) from our earlier study (Lopez-Poveda and Johannesen, 2012).

Figure 7(top) shows that pure OHC loss was rare and occurred mostly for low absolute thresholds (or, equivalently, small hearing losses). There were no cases of pure IHC loss, something not surprising considering that significant HL_IHC_ would probably make it impossible to measure a CT (Figure 1 in Lopez-Poveda and Johannesen, 2012) and Figure 7(top) only show results for cases with a CT. Most cases were in the shaded areas and thus were consistent with mixed IHC/OHC loss. The number of cases with mixed loss tended to increase with increasing absolute threshold (or hearing loss). Incidentally, the number of cases with mixed loss appeared somewhat larger at 2 kHz than at other frequencies. This may be somewhat artifactual due to our using the mean NH CT and absolute threshold at 4 kHz to estimate HL_OHC_ at 2 kHz. Any difference between the mean NH CTs at 2 and 4 kHz would bias the data horizontally and a difference between the mean NH absolute threshold at 2 and 4 kHz would bias the data vertically and thus might contribute to an apparent higher incidence of mixed IHC/OHC loss at 2 kHz.

#### From linear I/O curves

Linear I/O curves were assumed to be indicative of total gain loss. Hence, HL_OHC_ for these cases was set equal to the average cochlear gain for the NH reference group. The latter was estimated using Equation (2), and was equal to 35.2, 43.5, 42.7, 42.7, 42.7 dB at 0.5, 1, 2, 4, and 6 kHz. HL_IHC_ was then obtained using Equation (1).

Results for these cases are shown as blue crosses in the top panels of Figure 7. Clearly, the great majority of these cases were in the shaded area, hence were indicative of mixed OHC/IHC loss. In other words, for most of these cases, hearing loss was greater than the maximum possible mechanical cochlear gain loss (the gain loss of NH listeners), hence HL_IHC_ > 0 dB.

#### From compressive I/O curves without a compression threshold

As explained above, I/O curves that were either compressive straight lines (with slopes <0.75 dB/dB; Figure 5F), or that showed an RLT but not a CT (as in Figure 5H) were assumed indicative of IHC dysfunction. This is because any gain reduction will only affect the low-level linear portion of the I/O curve and IHC dysfunction may increase the BM response at detection threshold above the knee-point of the I/O curve (Figure 1B of Lopez-Poveda and Johannesen, 2012). Lopez-Poveda and Johannesen (2012) argued that for these cases Equation (1) does not hold, and that it is reasonable to assume that the audiometric loss can be fully explained in terms of inefficient IHC transduction combined with residual compression (see their Figure 1D). Therefore, we assumed that for these cases HL_TOTAL_ was equal to HL_IHC_.

This is not to say, however, that cochlear gain loss did not occur in these cases; we are saying that if cochlear gain loss did occur, it is unlikely that it contributed to the audiometric loss (see Figure 1D in Lopez-Poveda and Johannesen, 2012). Indeed, an estimate of (residual) gain was obtained as illustrated in Figure 5F or Figure 5H using Equation (2). Note that this gain estimate was almost certainly less than the actual residual gain because, due to IHC dysfunction, the measured compressive segment of the I/O was only a portion of the true compressive segment. Cochlear gain loss (HL_OHC_) was estimated by subtracting the obtained gain estimate from the reference gain for NH listeners (see the previous section). The bottom panels of Figure 7 illustrate residual gain for these cases. The left pointing arrows indicate that the actual HL_OHC_ was probably *smaller* than estimated, hence that symbols should be to the left of their position in the figure, and closer to the red-dotted line indicative of pure IHC loss. The figure reveals two important results: first, that most of these cases are indicative of mixed IHC and OHC dysfunction (indeed, mixed dysfunction appears more frequent for these cases than for I/O curves with a CT; compare the placement of blue triangles and circles in the bottom and top panels of Figure 7); and second, that for any given absolute threshold (or hearing loss), there were comparatively more cases with little gain loss (i.e., indicative of IHC dysfunction) at lower than at higher frequencies. In other words, low-frequency hearing loss is more likely related to IHC dysfunction than to cochlear gain loss.

### Across listener variability of HL_OHC_

**Figure 8:**
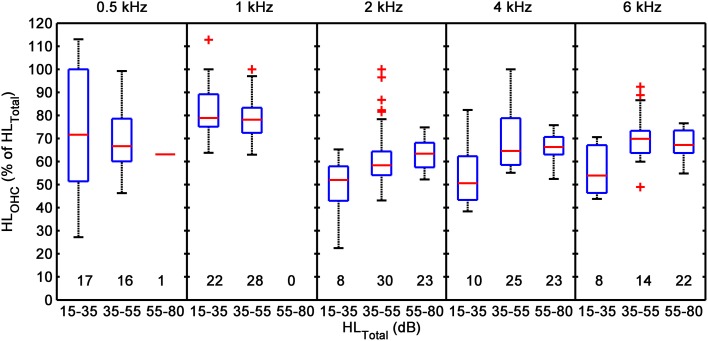
**Across-listener variability in the proportion of HL_TOTAL_ explained by HL_OHC_**. Each box plot illustrates distribution percentiles: the bottom and top lines in each box depict the first and third quartiles of the distribution; the band inside the box is the second quartile (the median); the lower and upper whiskers' ends depict the 1 and 99% percentiles. Crosses (+) depict cases outside the latter percentiles. Each panel is for a different test frequency. In each panel, distributions are given for HL_TOTAL_ ranges of 15–35, 35–55, and 55–80 dB. The numbers below each box plot indicate the number of cases (sample size) included in each distribution. The figure includes data for Type 1 and Type 2 I/O curves from the present participants and from the participants in our previous study.

Figure 6 suggests that HL_OHC_ accounted on average for 61–70% of HL_TOTAL_ but it also suggests that there was large across-listener variability. Figure 8 illustrates this variability more clearly by showing the distribution of HL_OHC_ for three different ranges of HL_TOTAL_: 15–35, 35–55, and 55–80 dB. Results are based on Type 1 and Type 2 I/O curves. At 2 kHz and above, HL_OHC_ tended to increase with increasing HL_TOTAL_, while at 0.5 and 1 kHz it decreased slightly or remained approximately constant. The main result from this figure is, however, that for a given frequency and hearing-loss range, HL_OHC_ was broadly distributed across cases. For example, based on data for 25 subjects, at 4 kHz and for a hearing-loss range of 35–55 dB, HL_OHC_ accounted for between 55 and 100% of HL_TOTAL_. [Note that the figure suggests that in a few cases with small losses, HL_OHC_ accounted for more than 100% of HL_TOTAL_. These were cases whose CTs were lower than the mean CT for the reference, NH group (i.e., cases below the diagonal line in Figure 7)].

### Prevalence of IHC and OHC dysfunction

The previous analyses have focused mostly on the relative contribution of HL_OHC_ and HL_IHC_ to HL_TOTAL_. The data may be alternatively analyzed with a focus on the type of hearing loss; that is, on how many data points fall in each of several regions depicted in Figure 7. To this end, Type 1 (CT present) and Type 2 (linear) I/O curves were split into two subcategories: “Pure OHC dysfunction,” when the audiometric loss could be entirely explained as loss of cochlear gain, that is, when HL_TOTAL_ ~ HL_OHC_ (points within the diagonal range in Figure 7); and “Mixed OHC/IHC dysfunction,” when the audiometric loss exceeded the cochlear gain loss (i.e., when HL_IHC_ > 0; points in the shaded area of Figure 7). For the reasons explained above, for Type 3 I/O curves, the absence of a CT was taken as indicative that the audiometric loss could be explained entirely in terms of IHC dysfunction (HL_TOTAL_ ~ HL_IHC_). As shown in the bottom panels of Figure 7, however, cochlear gain loss of uncertain extent still occurred in a majority of these cases even though it probably did not contribute to the audiometric loss. Therefore, Type 3 I/O curves were also regarded as indicative of mixed OHC/IHC dysfunction.

The top part of Table 3 gives the number of cases in each of these categories, and the bottom part of Table 3 the corresponding percentages. Note that the number of cases of Type 3 I/O curves decreased with increasing frequency, suggestive that IHC dysfunction was more determinant to audiometric loss at low frequencies than cochlear gain loss. Note also that the percentage of cases of pure OHC loss decreased with increasing frequency, while the percentage of cases of mixed loss increased with increasing frequency, and that the two percentages add up to 100%. Mixed OHC/IHC loss was significantly more frequent than pure OHC at all frequencies. The bottom part of Table 3 gives one additional percentage: “Total gain loss” refers to the total percentage of linear I/O curves, whether indicative of pure OHC dysfunction or mixed OHC/IHC dysfunction. The percentage of these cases increased with increasing frequency. Chi χ^2^ tests were used to test if the above described frequency trends were statistically significant. The null hypothesis was that for each I/O curve type, the frequency distribution followed the distribution of the total number of cases (i.e., the distribution in the line labeled as “Total” in the table).

**Table 3:** **Number of cases per I/O curve type and frequency (top) and percentage of cases per loss type (bottom)**.

		**Frequency (kHz)**					
**I/O curve type**	**Criterion**	**0.5**	**1**	**2**	**4**	**6**	***p***
Type 1 (CT present)	HL_TOTAL_ ~ HL_OHC_	5	26	1	3	2	<1e–6
	HL_IHC_, HL_OHC_ > 0	24	28	45	35	22	0.082
Type 2 (Linear)	HL_TOTAL_ ~ HL_OHC_	8	3	3	1	3	0.123^*^
	HL_IHC_, HL_OHC_ > 0	5	1	14	22	19	1e–6
Type 3 (CT absent with compression)	HL_TOTAL_ ~ HL_IHC_	25	15	7	3	5	3e–5
Total		67	73	70	64	51	
Loss type (%)	Total gain loss (linear I/O curve)	19.4	5.5	24.3	35.9	43.1	1.7e–4
	Pure OHC dysfun. (HL_TOTAL_ ~ HL_OHC_)	19.4	39.7	5.7	6.3	9.8	1e–6
	Mixed OHC/IHC dysfun. (HL_OHC_, HL_IHC_ > 0)	80.6	60.3	94.3	93.8	90.2	0.14

*p indicates significance levels for chi-squared tests. The asterisk indicates that the statistical test was not reliable because the number of cases was insufficient*.

### Verification and extension of model assumptions

**Figure 9:**
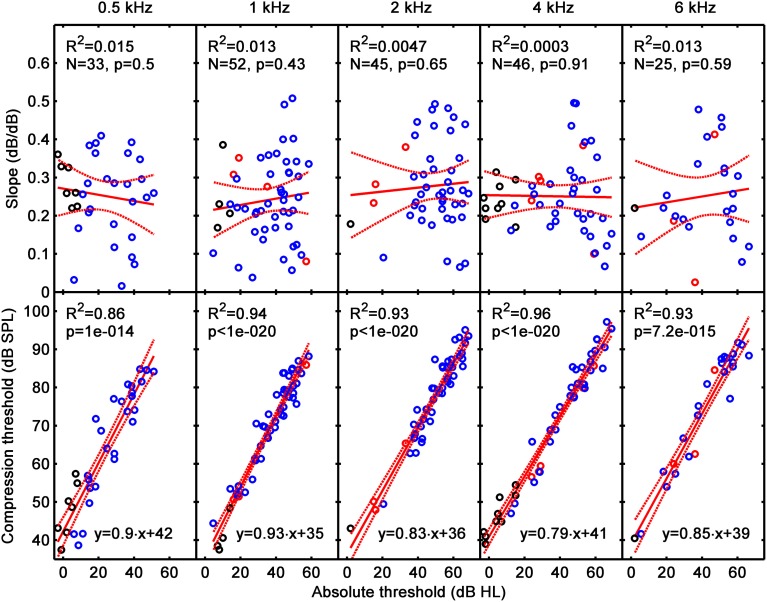
**Correlation of average slope (i.e., the slope of the I/O over the compressive segment) (top) and compression threshold (bottom) with absolute threshold**. Each column is for a different test frequency, as indicated by the column title. Results for the present listeners are depicted as blue symbols; results for NH listeners and for listeners with mild-to-moderate loss from our earlier study (Lopez-Poveda and Johannesen, 2012) are depicted by black and red symbols, respectively. Continuous and dotted lines depict mean linear regression and 5–95% confidence intervals of the regression functions, respectively. The linear regression function and related statistics are shown in the insets.

The present analysis was based on the hearing loss model of Plack et al. (2004) whereby OHC loss would reduce cochlear gain without significantly altering the amount of compression; that is, OHC loss would shift the low-level linear segment of the I/O curve without altering the slope of the compressive segment (Figure 7D of Plack et al., 2004). Their model was based on their observed lack of correlation between the compression exponent and absolute threshold accompanied by a strong negative correlation between gain and absolute threshold (their Figure 6). Their data was restricted to mild-to-moderate hearing losses and to a probe frequency of 4 kHz. Hence, one might object to the present analyses on the grounds that their model has not yet been corroborated for larger hearing losses or for the wider range of test frequencies used here. Our data, however, do support their model. Figure 9 shows that the CT, a parameter of the I/O curve directly related with cochlear gain, is positively and highly significantly correlated with absolute threshold (Figure 9, bottom) while the average slope over the compressive segment of the I/O curve (i.e., over the input level range from the CT to the RLT) is uncorrelated with absolute threshold (Figure 9, top). This supports the results of Plack et al. (2004) at 4 kHz, extends their model to greater hearing losses and to a wider frequency range from 0.5 to 6 kHz, and supports the validity of our approach.

## Discussion

The aim of the current study was threefold: (1) to assess to what extent the audiometric loss is due to a reduction in cochlear gain (or OHC dysfunction), and/or to an additional component, referred here to as IHC dysfunction; (2) to investigate the frequency distribution of the two potential contributions; and (3) to investigate the degree of variability of the two contributions across listeners. Our approach was based on the analysis of behaviorally inferred cochlear I/O curves, as we proposed elsewhere (Lopez-Poveda and Johannesen, 2012).

Regarding the first and second aims, results for Type 1 I/O curves (i.e., for curves with a CT) suggest that on average IHC and OHC dysfunction contribute 30–40 and 60–70% to the audiometric loss, respectively, and that these percentages hold approximately constant across the frequency range from 500 Hz to 6 kHz (Figure 6). Regarding the third aim, results suggest that the proportion of the audiometric loss attributed to cochlear gain loss can vary largely across listeners with similar hearing losses, without a clear frequency pattern (Figure 8). Cases for which audiometric thresholds could be explained exclusively in terms of IHC dysfunction (Type 3 I/O curves) or in terms of cochlear gain loss (points in the diagonal region of Figure 7) were comparatively more numerous at low than at high frequencies (Table 3). The large majority of cases, however, were consistent with mixed OHC/IHC dysfunction, even though in some of these cases (Type 3 I/O curves) cochlear gain loss was unlikely to contribute to the audiometric loss (Table 3). Total cochlear gain loss (i.e., linear I/O curves), occurred more frequently at high frequencies than at low frequencies (Table 3).

### Potential methodological sources of bias

#### On the accuracy of the TMC method for estimating I/O curves

In inferring I/O curves from TMCs, the assumption has been made that the post-mechanical rate of recovery from forward masking is independent of masker frequency and level (Nelson et al., 2001). Evidence exists, however, that for NH listeners the recovery rate is twice as fast for masker levels below around 83 dB SPL than for higher masker levels (Wojtczak and Oxenham, 2009). This level effect, however, does not occur for HI listeners (Wojtczak and Oxenham, 2010). There also exists evidence that the recovery rate might be slower at low (≤1 kHz) than at high probe frequencies (Stainsby and Moore, 2006), although this evidence is controversial (Lopez-Poveda and Alves-Pinto, 2008). Lopez-Poveda and Johannesen (2012) discussed that if these assumptions did not hold, Type 1 I/O curves (i.e., curves with a CT) would lead to larger HL_IHC_ and smaller HL_OHC_. In the present context, this means that if the assumptions were not valid, the contribution of HL_IHC_ to the total hearing loss might be higher than reported in Figure 6.

#### Ambiguity of linear I/O curves

Linear I/O curves have been assumed indicative of total cochlear gain loss. This assumption may be inaccurate sometimes. Assuming that cochlear I/O curves become linear at high input levels (something still controversial, Robles and Ruggero, 2001, pp. 1308–1309), for cases with substantial IHC dysfunction, the mechanical cochlear response at the probe detection threshold might be so much higher with respect to NH that only the high-level linear segment of the I/O curve can be measured (e.g., Figure 1D of Lopez-Poveda and Johannesen, 2012). Hence, linear I/O curves at high input levels may indicate two different things: total cochlear gain loss or substantial IHC dysfunction. It is not possible to distinguish between these two cases. Therefore, some of the cases presently classified as “total cochlear gain loss” (or total OHC dysfunction) may actually reflect substantial IHC dysfunction.

An arbitrary slope criterion of 0.75 dB/dB has been used to separate Type 2 from Type 3 I/O curves. A sensitivity analysis was done to test to what extent results depended on the slope criterion value and we found that only five out of the 325 I/O curves would change type if the slope criterion were varied from 0.6 to 1 dB/dB. Therefore, I/O curve classification seems rather insensitive to slope criterion within these limits.

#### The impact of using a mean linear-reference TMC for some cases

A linear reference TMC could not be measured for eight participants (four of them from our previous study) because their hearing losses at the linear reference probe frequencies (Table 1) were so high that masker levels would have exceeded the maximum output level of our system. I/O curves for these cases were inferred using a mean linear reference TMC from all other subjects (see Methods). It is unlikely that this methodological difference affected the main results. First, CTs inferred using the mean linear reference TMC were within 5-dB of corresponding estimates inferred using the variant TMC method of Lopez-Poveda and Alves-Pinto (2008), a method that does not require a linear reference TMC (results not shown). Second, the number of I/O curves inferred using a mean linear reference TMC was only a very small fraction of the total number of I/O curves used in the present study.

#### Cochlear gain for normal hearing listeners and total OHC loss

Linear I/O curves were regarded as indicative of total cochlear gain loss (Figures 4, 5). For these cases, HL_OHC_ was set equal to the mean cochlear gain of the reference, NH group. If the latter were inaccurate, this could have affected the present estimates of HL_OHC_ (i.e., the number and position of blue crosses in Figure 7). Gain for the NH group was calculated as described in section I/O Curve Analyses and Taxonomy and one might argue that this method underestimated gain for those NH I/O curves with absent CT or RLT; that is, for I/O cures that were still compressive at the lowest or the highest input levels in the I/O curve. The present NH gain values at high frequencies, however, compare well with previously reported values inferred using different psychoacoustical methods and with values inferred from direct BM recordings. For example, at 4 kHz, mean gain was 42.7 dB hence comparable to the value (43.5 dB) reported by Plack et al. (2004). Plack et al. estimated gain as the difference between the masker levels of the linear-reference and on-frequency TMCs for the shortest gap, while gain was defined here as the sensitivity difference for low and high input levels (see Ruggero et al., 1997 for a discussion of different gain definitions). Gain for the present NH group would have been 48.9 dB had it been calculated using the definition of Plack et al. (2004), hence slightly higher than the value of Plack et al. The present NH gain compares well also with the value (35 dB at 6 kHz) that would be obtained from the I/O curves in Figure 2 of Oxenham and Plack (1997) that were inferred using a different psychoacoustical method known as growth of forward masking. Also, the present NH gain values at 4 kHz are within the value range suggested by direct basal BM recordings (range = 19–62 dB; median = 40 dB; mean = 38 dB; Table 1 of Robles and Ruggero, 2001). Altogether, this suggests that the present high-frequency NH gain values were reasonable.

Direct BM recording in animals suggest that cochlear gain is less for apical than for basal BM regions although it is possible that the difference is partly due to damage of apical cochlear mechanics during experimental recordings. For example, the change of chinchilla BM sensitivity at the characteristic frequency between low and high input levels is 10–20 dB at 500–800 Hz compared to 50 dB at 8–9 kHz (Tables 2, 3 in Robles and Ruggero, 2001). Previous psychoacoustical reports in humans using other methods and assumptions also suggest less gain at low frequencies but do not provide quantitative estimates (Plack et al., 2008). Gain estimates for the present NH group were 35.2 dB at 500 Hz and 42.7 dB at 4 kHz. The frequency trend in the present results is thus qualitatively consistent with direct BM observations, and quantitative differences might be due to differences in cochlear tonotopic mappings across species. If, however, the post-mechanical rate of recovery from forward masking were after all faster at lower frequencies (see previous sections), then cochlear gain would be smaller than reported here and the pattern of results would become more consistent with the animal data.

In summary, the NH gain values used here to quantify HL_OHC_ for cases of total OHC loss (linear I/O curves) seem reasonable at high frequencies but are less certain at low frequencies.

Incidentally, it is noteworthy that the present NH gain increased from 35.2 dB at 500 Hz to 43.5 dB at 1 kHz (unpaired, equal variance, *t*-test, *p* = 0.014) and then gain remained constant at higher frequencies (42.7 dB at 4 kHz). This pattern differed slightly from that reported by (Johannesen and Lopez-Poveda, 2008), from where some of the present NH data were taken. Indeed, in that study, gain increased gradually with increasing frequency from 37 dB at 500 Hz to 55 dB at 4 kHz (see their Figure 11A). This discrepancy is almost certainly due to methodological differences. First, the two studies used different definitions of gain; Johannesen and Lopez-Poveda (2008) calculated gain as the difference between the RLT and CT. Second, the present NH data combined data from the 10 participants that took part in the study of Johannesen and Lopez-Poveda (2008) plus data for five more NH participants from Lopez-Poveda and Johannesen (2009); the latter contributed data particularly at 0.5 and 1 kHz. Third, Johannesen and Lopez-Poveda (2008) fitted their I/O curves with a third-order polynomial, which “forces” an RLT when a CT is present because the slopes of a third-order polynomial are identical below and above its inflection point. Indeed, fewer of the I/O curves from the study of Johannesen and Lopez-Poveda (2008) retained an RLT when they were re-analyzed using the present fitting approach.

#### The influence of conductive hearing loss on the results

Participants were controlled for conductive hearing loss. Nonetheless, their air-bone gaps could have differed by ≤15 dB at one frequency and/or ≤10 dB at any other frequency (see Methods). Small conductive losses might have increased probe absolute threshold and hence TMC masker levels by an amount equal to the conductive loss at the corresponding probe frequencies. The influence on the inferred I/O curve would be an upward vertical shift of the I/O curve equal to the conductive loss at the frequency of the linear reference probe and a rightward horizontal shift equal to the conductive loss at the frequency of the on-frequency masker. The CT would be affected only by the horizontal shift. Therefore, conductive loss at the particular frequency might lead to an overestimate of HL_OHC_ at that frequency. Pearson's correlation between HL_OHC_ and air-bone gap was significant only at 1 kHz and indicated decreasing HL_OHC_ for increasing air-bone gap. The direction of the effect was therefore opposite to the presumed effect of conductive hearing loss on HL_OHC_ and hence we concluded that conductive loss was unlikely to affect mean HL_OHC_ estimates in Figure 6.

#### The potential influence of dead regions on the results

A “dead region” is “a region in the cochlea where the IHCs and/or neurons are functioning so poorly that a tone which produces peak BM vibration in that region is detected via an adjacent region where the IHCs and/or neurons are functioning more efficiently” (p. 272 in Moore, 2007). In principle, dead regions could affect TMC measures as the probe presented in a dead region would be detected at a cochlear place removed from the probe place: e.g., at a place where the on-frequency masker might be subject to a compression regime different from compression at the normal probe place. For example, if the 4-kHz cochlear region was dead, a 4-kHz probe might be detected at the 2-kHz cochlear region where a 1.6-kHz (off-frequency) masker, which is typically regarded as a linear-reference condition, might be actually subject to significant compression.

Dead regions occur almost always for hearing losses above ~60 dB HL (Table 1 in Vinay and Moore, 2007) and the present listeners were roughly selected to have hearing losses <80 dB HL to be able to measure TMCs for a majority of test frequencies (Figure 1). Despite this, TMCs could not be measured for the higher losses. Of the 325 measured I/O curves, the number that may have been affected by dead regions can be roughly estimated from the data in Table 1 of Vinay and Moore (2007) (note that their data goes to 4 kHz only and we have assumed that the incidence of dead regions is identical at 4 and 6 kHz). Our analysis revealed that the expected incidence of dead regions was one, two and two at 2, 4, and 6 kHz, respectively. These numbers are so low that they are unlikely to have biased the reported HL_OHC_ and HL_IHC_.

### Comparison with earlier studies

Based on our analysis of Type 1 I/O curves, we have shown that HL_OHC_ is 60–70% of HL_TOTAL_ across the frequency range from 0.5 to 6 kHz. This number is roughly consistent with that reported by earlier studies for more restricted frequency ranges, mostly at 4 kHz (Plack et al., 2004; Lopez-Poveda and Johannesen, 2012). It is, however, slightly lower than the 80–90% value reported elsewhere based on loudness models (Moore and Glasberg, 1997). Jürgens et al. (2011) showed that the two approaches (loudness model and TMCs) should give similar results. Therefore, the reason for this difference is uncertain.

We have also shown that even though the percentage of cases for which HL_IHC_ accounts entirely for HL_TOTAL_ (the percentage of Type 3 I/O curves) or the percentage of cases for which HL_OHC_ ~ HL_TOTAL_ (the percentage of pure OHC dysfunction) are small, they are both larger for frequencies ≤1 kHz and decrease with increasing frequency (Table 3). To the best of the authors' knowledge, these trends have not been reported explicitly before, possibly due to the use of small sample sizes in earlier studies, but are not without precedent. For example, Moore and Glasberg (1997) used a model of loudness growth to estimate HL_IHC_ and found that it increased with decreasing frequencies for three listeners. Likewise, Jepsen and Dau (2011) reported greater HL_IHC_ at lower frequencies for a few subjects, although their average results were still consistent with the common notion that the most typical functional deficit is the loss of mechanical gain in the cochlear base.

An important distinction between the present and earlier analyses is that here, HL_IHC_ and HL_OHC_ were not always regarded as mutually exclusive, additive contributions to HL_TOTAL_. Instead, the possibility has been contemplated that Equation (1) does not hold for cases where IHC dysfunction is so significant that it makes it impossible to measure a CT. In these cases, it was assumed that HL_TOTAL_ may be explained fully in terms of HL_IHC_ even though concomitant cochlear gain loss did probably occurred (Figure 7, bottom).

### Structure-function relationships

Great care must be exercised at establishing a direct link between the behavioral deficits seen here (audiometric loss and cochlear gain loss) and hair cell pathophysiology in humans. Discussing potential relationships might be nonetheless useful.

We have shown that for a large percentage of cases (Type 1 I/O curves), 60–70% of HL_TOTAL_ is due to HL_OHC_ and 30–40% is due to HL_IHC_, and that these percentages are roughly constant across frequencies (Figure 6). It would be probably wrong to conclude that this implies identical physical damage to OHCs and IHCs along the cochlear length. First, when physical hair cell damage occurs (e.g., after noise exposure), it is typically greater in the cochlear base than in the apex (Møller, 2000). Second, the median age of the present participants was 61 years, hence for most of them the cause of hearing loss was probably presbycusis. Presbycusis is associated with a reduction of the endocochlear potential that causes high-frequency hearing loss (Schmiedt et al., 2002). This high-frequency loss is almost certainly due to concomitant, combined IHC and OHC dysfunction. A given reduction of the endocochlear potential causes greater loss of cochlear gain in the cochlear base than in the apex (Figure 8 of Saremi and Stenfelt, 2013), and a reduced response in the IHCs (Meddis et al., 2010; Panda et al., 2014). The present results for Type 1 I/O curves are consistent with concomitant IHC and OHC dysfunction characteristic of metabolic presbycusis and less so with the alternative and perhaps prevailing view that high-frequency loss is due to greater anatomical loss or damage of basal OHCs.

We have also shown, however, that the percentage of Type 2, linear I/O curves increases with increasing test frequency (Figure 7-top and Table 3). If metabolic presbycusis linearized cochlear responses (Saremi and Stenfelt, 2013), this might be indicative that metabolic presbycusis reduces the endocochlear potential more in the cochlear base than in the apex, something unlikely. A more parsimonious explanation for the higher percentage of linear I/O curves at high frequencies would be that they are actually due to severe physical OHC loss or damage. The latter explanation would be consistent with the prevailing view that physical OHC damage is greater in the cochlear base than in the apex (Møller, 2000).

Lastly, we have also shown that the percentage of Type 3 I/O curves is greatest for test frequencies ≤1 kHz and decreases with increasing frequency. This trend of more frequent IHC dysfunction at apical sites remains intriguing. A few studies have reported similar trends. For example, apical IHCs were found to be more labile than basal IHCs in guinea pigs treated with polypeptide antibiotics (Kohonen, 1965). Similarly, after administration of tobramycin, IHCs were found to be normal in the base but completely damaged in the apex whereas the OHCs were found to be normal in the apex and damaged in the base (Aran et al., 1982). Therefore, some Type 3 I/O curves might be indicative of antibiotic-induced hearing loss.

Unfortunately, confirmation of these conjectures was not possible due to the lack of accurate information regarding the etiology of hearing loss for the present participants.

## Conclusions

With regard to the contribution of IHC and OHC dysfunction to the audiometric loss, the main conclusions are:

1. For cases where a CT is present, IHC and OHC dysfunction contribute on average to 30–40 and 60–70% to the total audiometric loss, and these contributions are approximately constant across the frequency range from 0.5 to 6 kHz.
2. The individual variability of the relative contributions of IHC and OHC dysfunction to the audiometric loss is, however, large particularly at low frequencies or mild-to-moderate hearing losses.

With regard to the incidence of dysfunction types, the main conclusions are:

3. The large majority of cases suffer from mixed IHC and OHC dysfunction, even though in some cases with presumably substantial IHC dysfunction, any concomitant OHC dysfunction does not contribute to the audiometric loss.
4. The percentage of cases for which the audiometric loss can be explained exclusively in terms of cochlear gain loss or of inefficient IHC processes (i.e., cases of pure OHC or IHC dysfunction, respectively) is higher at frequencies ≤1 kHz and decreases gradually with increasing frequency.
5. The percentage of cases suffering from total cochlear gain loss (i.e., linear I/O curves) increases gradually with increasing frequency.

Overall, the present results undermine the common view that high-frequency loss is typically due to greater physical damage of basal OHCs, and suggest that in a large percentage of cases, it is due to a common mechanism that concomitantly affects IHCs and OHCs, possibly reduced endocochlear potential. They further suggest that IHC processes may be more labile in the apex than in the base and/or that IHC dysfunction may have a greater impact on auditory threshold than cochlear gain loss at low frequencies.

### Conflict of interest statement

The authors declare that the research was conducted in the absence of any commercial or financial relationships that could be construed as a potential conflict of interest.

## Footnotes

^1^Because each TMC was measured at least three times, we could assess the variance in *L_A_*, *L_B_*, *L_C_*, and *L_D_*, hence the gain variance (Equation 2). A Student's *t*-test was then used to verify if the mean gain estimate was statistically greater than zero at the 5% significance level.
